# A three‐tiered integrative analysis of transcriptional data reveals the shared pathways related to heart failure from different aetiologies

**DOI:** 10.1111/jcmm.15544

**Published:** 2020-07-08

**Authors:** Zhenhong Jiang, Ninghong Guo, Kui Hong

**Affiliations:** ^1^ The Jiangxi Key Laboratory of Molecular Medicine Nanchang China; ^2^ Department of Cardiovascular Medicine The Second Affiliated Hospital of Nanchang University Nanchang China

**Keywords:** gene set enrichment analysis, heart failure, pathway, transcriptional data

## Abstract

Heart failure (HF) is the end stage of most heart disease cases and can be initiated from multiple aetiologies. However, whether the molecular basis of HF has a commonality between different aetiologies has not been elucidated. To address this lack, we performed a three‐tiered analysis by integrating transcriptional data and pathway information to explore the commonalities of HF from different aetiologies. First, through differential expression analysis, we obtained 111 genes that were frequently differentially expressed in HF from 11 different aetiologies. Several genes, such as *NPPA* and *NPPB*, are early and accurate biomarkers for HF. We also provided candidates for further experimental verification, such as *SERPINA3* and *STAT4*. Then, using gene set enrichment analysis, we successfully identified 19 frequently dysregulated pathways. In particular, we found that pathways related to immune system signalling, the extracellular matrix and metabolism were critical in the development of HF. Finally, we successfully acquired 241 regulatory relationships between 64 transcriptional factors (TFs) and 17 frequently dysregulated pathways by integrating a regulatory network, and some of the identified TFs have already been proven to play important roles in HF. Taken together, the three‐tiered analysis of HF provided a systems biology perspective on HF and emphasized the molecular commonality of HF from different aetiologies.

## 1 INTRODUCTION

Heart failure (HF) is a global pandemic heart disease characterized by an inadequate systemic perfusion to meet the body's metabolic demands. It is estimated to affect approximately 26 million people worldwide and results in a heavy burden on the economy and healthcare system.^1^ Moreover, the prevalence of HF is expected to increase with population growth and ageing.^2^ HF is a complex disease influenced by environmental and genetic factors. A wide range of conditions can lead to HF, such as hereditary defects, cardiovascular diseases and systemic diseases, which indicates molecular commonalities between HF resulting from different aetiologies. Thus, exploring the commonalities will help achieve better understanding of the aetiology of HF.

The rapid development of high‐throughput ‘omics’ technologies (such as DNA microarrays and next‐generation sequencing) has resulted in the increasing availability of transcriptional data.^3^ The availability of these data for HF provides a good opportunity to employ computational systems biology approaches to advance our understanding of the mechanisms underlying the development of HF.^4^, ^5^ Studies based on transcriptional and interactome data have identified 60 common functional modules related to HF^6^ and analysed the differences in the pathogenesis of HF arising from different aetiologies.^7^ By examining the expression profiling of miRNAs in failing human hearts, Zhu et al identified miR‐340 as a key miRNA contributing to the progression of HF.^8^ By integrating miRNA‐target interactions and differentially expressed genes/lncRNAs, a recent study investigated the function of lnRNAs in HF and identified some lncRNAs that were verified to show a strong diagnostic power for HF.^9^ Although some studies have been carried out to decipher the molecular mechanisms of HF based on transcriptional data, a systematic research study that comparatively analyses HF from different aetiologies through integration of vastly available omics data and curated pathways has not been performed.

**Figure 1:**
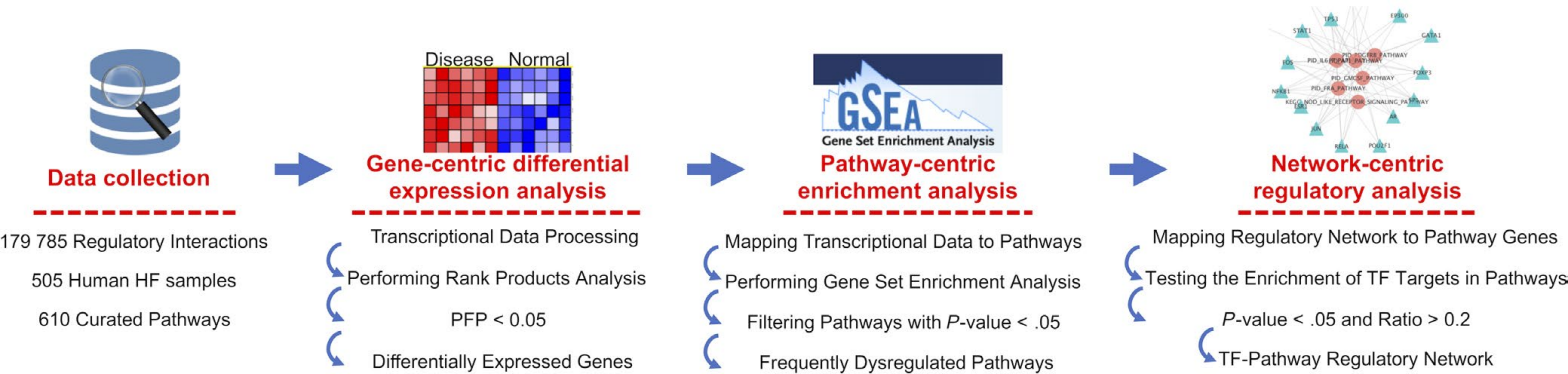
Overview of the workflow of the three‐tiered data analysis. First, we collected HF‐related transcriptional data from the GEO database, regulatory network data from RegNetwork and HTRIdb, and curated pathways from MSigDB. Then, we performed three‐tiered data analysis: gene‐centric differential expression analysis, pathway‐centric enrichment analysis and network‐centric regulatory analysis

To address this issue, we perform a three‐tiered data analysis (Figure 1). At the gene level, we globally analysed the gene expression of HF from 11 different aetiologies. At the pathway level, we comparatively analysed the expression of 610 curated pathways and identified 19 frequently dysregulated pathways in HF from 11 different aetiologies. Finally, by integrating the regulatory network, we identified several transcriptional factors (TFs) regulating the expression of the 19 frequently dysregulated pathways. Taken together, our work may provide new insights to better understand HF from different aetiologies.

## 2 MATERIALS AND METHODS

### 2.1 Overview of the data analysis procedure

To understand the molecular commonalities of HF from different aetiologies, we performed a three‐tiered data analysis (Figure 1). First, we started the analysis at the gene level, in which differentially expressed genes (DEGs) were inferred for each disease. Then, we extended our analysis to the pathway level, where we identified frequently dysregulated pathways across HF from different aetiologies. Finally, by integrating the regulatory network, we identified TFs regulating the expression of the frequently dysregulated pathways.

### 2.2 Data collection and pre‐processing

Transcriptional data related to HF resulting from 11 different aetiologies were collected from the GEO (Gene Expression Omnibus) database.^10^ Normalized data were directly downloaded from the GEO database. Probe sets were mapped to their corresponding gene symbol according to the annotation files from GEO, and replicated probes of the same gene were averaged. Curated pathways were gathered from the KEGG pathway,^11^ Reactome pathways,^12^ BioCarta pathways, Pathway Interaction Database,^13^ Sigma‐Aldrich gene sets, Signal Transduction KE gene sets, Signaling Gateway gene sets and SuperArray gene sets from the molecular signatures database (MSigDB, v6.1).^14^ The curated pathways were downloaded in GMT format. Of the available pathways, we used those from C2:CP (canonical pathways). After excluding pathways that were too large or too small (>300 genes or <5 genes, respectively) and removing overlapping pathways (overlap ratio > 0.8), 610 pathways were kept for further analyses.

### 2.3 Differential expression analysis

DEGs between disease samples and the corresponding control samples were inferred using the function RankProducts in the BioConductor package RankProd.^15^ For each disease, the fold changes (FCs) of genes between the disease and control samples were first translated to the ranks of genes. Then, the combined rank of each gene from multiple comparisons was defined as the rank product. Independent permutated expression data were used to calculate the null density of the rank product and to determine the p‐value and the percentage of false‐positive predictions (pfp) associated with each gene. Finally, genes with pfp values less than 0.05 were defined as differentially expressed.

### 2.4 Machine learning analysis

Random forest (RF) classification models were built using the ‘randomForest’ package (https://cran.r‐project.org/web/packages/randomForest/) in R with genes (features) in columns and samples in rows. We utilized a 10‐fold cross‐validation procedure to assess the performance of the classification models. Samples were randomly partitioned into 10 parts with approximately equal number of samples. Nine parts were used to train the RF classifier, and the remaining one part was used to test the performance. The value of the area under the curve (AUC) from the receiver operating characteristic (ROC) curve was used to assess the prediction accuracy of the RF model. The higher AUC value, which ranges from 0 to 1, indicates better prediction performance. After all samples have been used as the testing set, the predicted values were imported into the R package PRROC to visualize the ROC curves.^16^

### 2.5 Gene ontology enrichment analysis

Gene ontology (GO) enrichment analysis for DEGs was performed with BiNGO (version 3.03), a plugin in Cytoscape. Using the whole annotation of human genes as the reference set, GO terms with Benjamini‐Hochberg (BH)–adjusted *P*‐values less than 0.05 were extracted as significantly enriched.

### 2.6 Gene set enrichment analysis

The pre‐ranked gene set enrichment analysis (GSEA) tool^17^ [GSEA PreRanked (1000 permutations, minimum term size of 5, maximum term size of 300)] was used to determine whether the curated pathways exhibited statistically significant, concordant difference between HF and normal tissue samples. Briefly, genes were first ranked based on their FCs between disease samples and the corresponding normal samples. Then, ranked genes were used as the input for GSEA PreRanked. Finally, curated pathways with *P*‐values less than 0.05 were identified as significant.

### 2.7 TF‐pathway regulation analysis

To identify TFs regulating the dysregulated pathway, we first obtained TF‐target regulatory relationships from two databases, RegNetwork^18^ and HTRIdb.^19^ HTRIdb is an open access database that stores experimentally verified human transcriptional regulation interactions. RegNetwork integrates curated regulatory interactions among transcription factors, microRNAs (miRNAs) and target genes from various databases and potential regulatory relationships based on transcription factor binding sites. Then, for each TF and dysregulated pathway, Fisher's exact test was used to test the enrichment of TF targets in the dysregulated pathway, and a *P*‐value was obtained. We also calculated the proportion of TF targets in the pathway and obtained a ratio. Finally, the TF was predicted to regulate the dysregulated pathways when the BH‐corrected *P*‐value was less than 0.05 and the ratio was larger than 0.2.

## 3 RESULTS

### 3.1 Overview of gene expression in HF from different aetiologies

**Figure 2:**
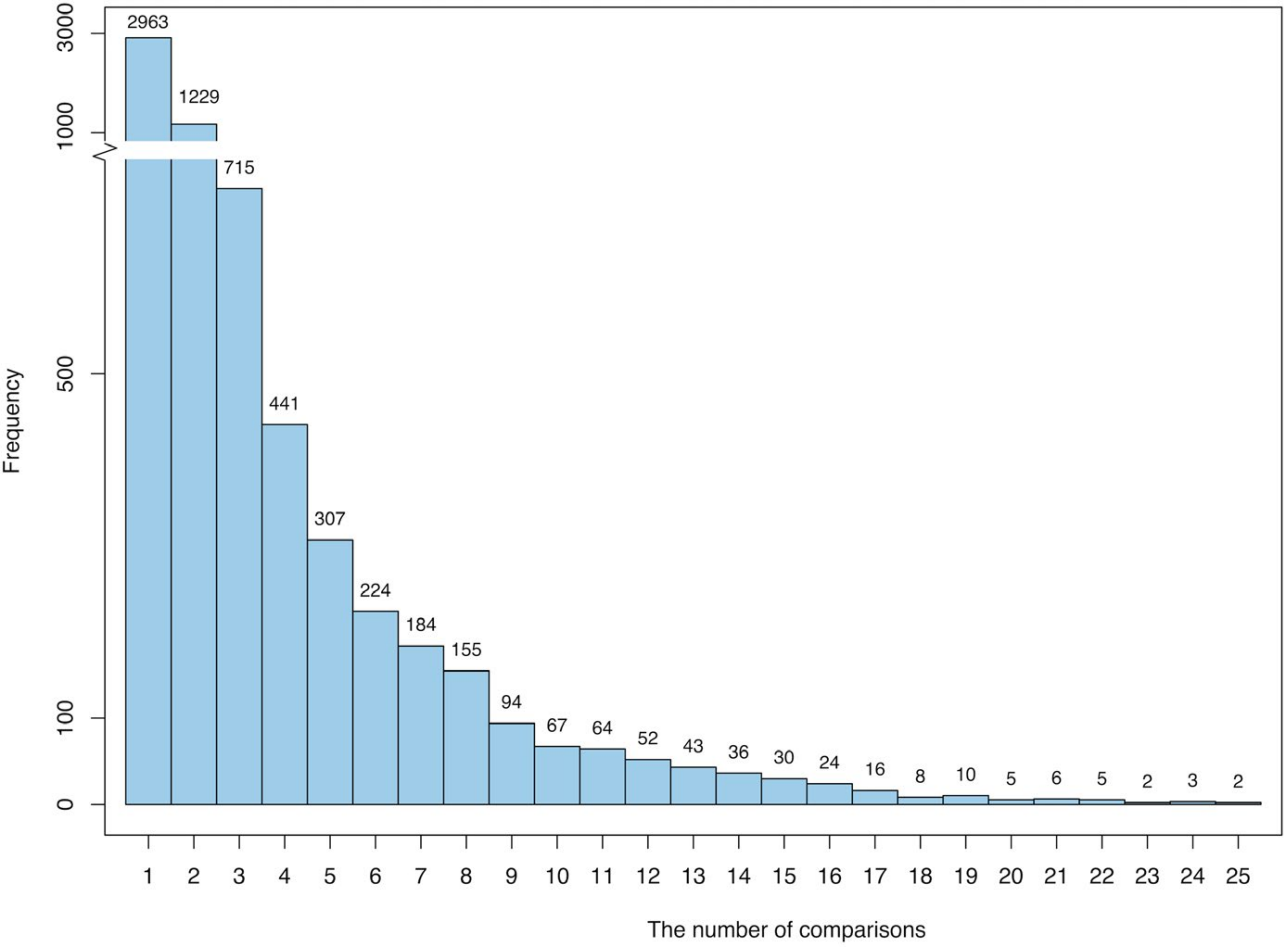
A total of 6,685 DEGs were shared between different numbers of disease conditions. The *x*‐axis shows the number of comparisons, and the *y*‐axis represents the number of DEGs. The number above each histogram refers to the number of DEGs that were shared under the given number of comparisons

To understand how genes are expressed during HF, we collected 505 samples from 11 microarray studies measuring gene expression during HF from 11 different aetiologies from the GEO database^10^ (Table 1, Table S1). Among the 505 collected samples, 414 samples detected gene expression in patients with HF, and the remaining 91 samples were from controls. The DEGs between the disease samples and their corresponding control samples were inferred using the RankProd package, which was developed from the rank product method.^15^ Rank product is a nonparametric statistical method for identifying DEGs (up‐regulated or down‐regulated) based on the estimated pfp. By keeping genes with a pfp of less than 0.05, we obtained 6685 DEGs that were differentially expressed in at least one of the 25 comparisons (Table S2; see Materials and Methods section for details). As shown in Figure 2, only approximately 3.6% (242/6685) of the DEGs were differentially expressed in more than 12 comparisons. This indicated that only a small fraction of DEGs were frequently differentially expressed during the progression of HF from different aetiologies.

**Table 1:** Transcriptional data used in this work

GEO_ID	Tissue	#DCM	#ICM	#IDCM	#DHF	#ND‐HF	#VCM	#ARVC	#NICM	#HCM	#FDCM	#PPCM	#Control
GSE76701	Left ventricle		4										4
GSE21610	Left ventricle	21	9										8
GSE26887	Left ventricle				7	12							5
GSE3585	Left ventricle	7											5
GSE42955	Left ventricle	12	12										5
GSE29819	Left ventricle	7						6					6
GSE29819	Right ventricle	7						6					6
GSE1869	Heart		10						21				6
GSE5406	Heart		108	86									16
GSE52601	left ventricular	4	4										4
GSE1145	Left ventricle		20	15			7			5	5	4	11
GSE16499	Left Ventricle		15										15

Abbreviations: DCM: Dilated cardiomyopathy; ICM: ischaemic cardiomyopathy; IDCM: idiopathic dilated cardiomyopathy; DHF: diabetic heart failure; ND‐HF: non‐diabetic heart failure; VCM: viral cardiomyopathy; ARVC: arrhythmogenic right ventricular cardiomyopathy; NICM: non‐ischaemic cardiomyopathy; HCM: hypertrophic cardiomyopathy; FDCM: familial dilated cardiomyopathy; PPCM: postpartum cardiomyopathy.

### 3.2 Identification of a common gene expression signature of HFs from different aetiologies

To identify the genes that were frequently differentially expressed (named frequently differentially expressed genes, FDEGs) during HF from different aetiologies, we selected genes that were differentially expressed in more than 60% (15/25) of comparisons and obtained 111 such genes (Table S2). Approximately 60% (67/111) of FDEGs were classified as up‐regulated since these genes were generally more up‐regulated in all comparisons, whereas the remaining 44 genes were classified as down‐regulated. Next, we investigated whether the two conditions (HF and normal control) could be successfully discriminated using the FDEGs by employing a RF classifier. By removing 16 FDEGs which were not detected in all 505 samples, the expression of 95 FDEGs was used as the feature. The AUC value from the ROC curve was used to evaluate the prediction accuracy of the RF classifier, and we found that FDEGs could classify HF and normal control samples with AUC 0.968 (Figure S1; see the Materials and Methods section for detail). However, when we randomly selected the equal number of genes (95) from the gene sets detected on the microarray to construct the RF classifier and repeated this process 1000 times, the average AUC was only 0.912, which was significantly lower than the AUC value of RF classifier constructed from the 95 FDEGs (Student's *t* test, *P*‐value <2.2 × 10^−16^). This result further confirmed the presence of multiple FDEGs among HF from different aetiologies was due to the shared molecular mechanisms.

**Figure 3:**
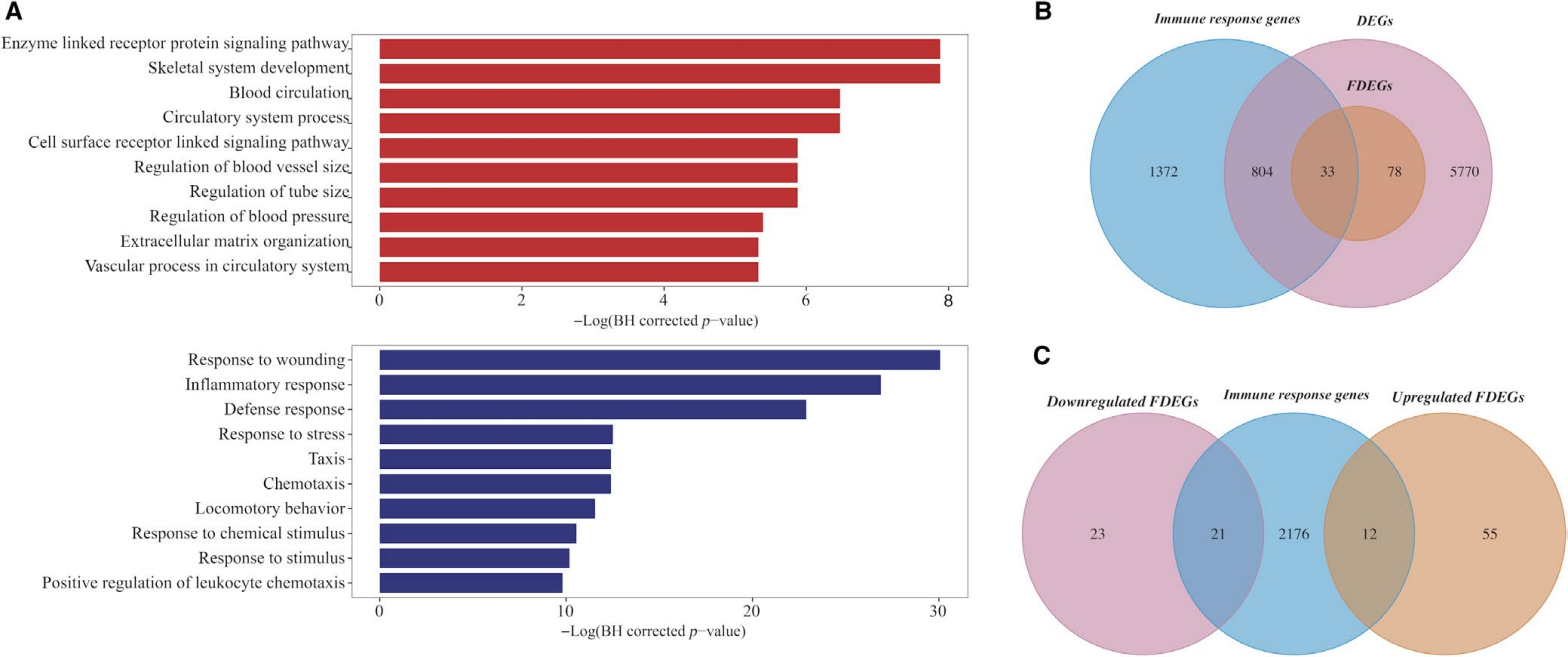
Annotation results for FDEGs. A, Top 10 annotation results for 67 up‐regulated FDEGs (red bar) and 44 down‐regulated FDEGs (blue bar). Annotation analysis was performed with BiNGO (Version 3.03). B, Three‐way Venn diagram representing the overlap among 2209 immune response genes, 6685 DEGs and 111 FDEGs. C, Three‐way Venn diagram representing the overlap among 2209 immune response genes, 44 up‐regulated FDEGs and 67 down‐regulated FDEGs

GO enrichment analysis showed that up‐regulated and down‐regulated FDEGs were enriched in different biological processes (BP) (Table S3, Figure 3A). The top 3 enriched GO BP terms for up‐regulated FDEGs were ‘enzyme‐linked receptor protein signalling pathway’ [hypergeometric test, BH‐corrected *P* = 3.77 × 10^−4^], ‘skeletal system development’ (hypergeometric test, BH‐corrected *P* = 3.77 × 10^−4^) and ‘blood circulation’ (hypergeometric test, BH‐corrected *P* = 1.54 × 10^−3^). For down‐regulated FDEGs, ‘response to wounding’ (hypergeometric test, BH‐corrected *P* = 8.72 × 10^−14^), ‘inflammatory response’ (hypergeometric test, BH‐corrected *P* = 2.10 × 10^−12^) and ‘defence response’ (hypergeometric test, BH‐corrected *P* = 1.15 × 10^−10^) were the top 3 enriched GO terms. Table 2 lists the top 20 FDEGs, which were most frequently differentially expressed in HF from 11 different aetiologies, including 12 up‐regulated genes and 8 down‐regulated genes.

**Table 2:** The top 20 FDEGs

Gene symbol	Full name	#Up	#Down
*SERPINA3*	*Serpin family A member 3*	0	25
***FCN3***	*Ficolin 3*	0	25
***NPPA***	*Natriuretic peptide A*	24	0
*CCL2*	*C‐C motif chemokine ligand 2*	2	22
***PLA2G2A***	*Phospholipase A2 group IIA*	1	23
***NPPB***	*Natriuretic peptide B*	17	6
*MYH6*	*Myosin heavy chain 6*	0	23
*OGN*	*Osteoglycin*	22	0
*USP9Y*	*Ubiquitin‐specific peptidase 9*	18	4
*EIF1AY*	*Eukaryotic translation initiation factor 1A, Y‐linked*	17	5
*RPS4Y1*	*Ribosomal protein S4, Y‐linked 1*	16	6
***S100A8***	*S100 calcium binding protein A8*	0	22
*SFRP4*	*Secreted frizzled‐related protein 4*	21	0
*ASPN*	*Asporin*	21	0
***FRZB***	*Frizzled‐related protein*	21	0
*STAT4*	*Signal transducer and activator of transcription 4*	20	1
*ANKRD2*	*Ankyrin repeat domain 2*	2	19
*RARRES1*	*Retinoic acid receptor responder 1*	0	21
*THBS4*	*Thrombospondin 4*	20	0
*NAP1L3*	*Nucleosome assembly protein 1 like 3*	20	0

Note

‘#Up’ and ‘#Down’ represent the number of disease conditions in which the corresponding genes are up‐regulated and down‐regulated, respectively. Genes with confirmed roles in HF are marked in bold.

For the top 20 FDEGs, six genes, namely natriuretic peptide A (*NPPA*), natriuretic peptide B (*NPPB*), Ficolin 3 (*FCN3*), phospholipase A2 group IIA (*PLA2G2A*), S100 calcium binding protein A8 (*S100A8*) and frizzled‐related protein (*FRZB*), were already shown to play important roles in HF or cardiovascular disease. *NPPA*, *NPPB* and *FRZB* are recognized as biomarkers for HF,^20^, ^21^ and *PLA2G2A* is a biomarker for cardiovascular disease.^22^ *FCN3* is a recognition molecule in the lectin pathway, and the decreased concentration of *FCN3* in serum has already been associated with the pathophysiology of HF.^23^ The other FDEGs (such as *STAT4* and *SERPINA3*) with undefined roles in HF were good candidates for further experimental verification as these genes were frequently differentially expressed in HF from different aetiologies.

### 3.3 Suppressed immune responses in HF

GO annotation analysis showed that the GO term ‘defence response’ was significantly enriched in down‐regulated FDEGs. We wondered whether FDEGs were significantly enriched in defence genes. Thus, we first obtained 2209 genes that were involved in the immune response from two databases, namely InnateDB^24^ and the Immunogenetic Related Information Source (IRIS).^25^ After removing genes without expression values from 2209 immune response genes, 2056 genes were kept for further analysis. Statistical analysis showed that FDEGs were significantly enriched in these immune‐related genes (Fisher's exact test, *P* = 1.01 × 10^−06^; Figure 3B). Further investigation showed that immune‐related FDEGs were significantly enriched in down‐regulated FDEGs (Table S2, Fisher's exact test, *P* = 1.04 × 10^−08^) rather than in up‐regulated FDEGs (Fisher's exact test, *P* = 0.13; Figure 3C). Moreover, the top 5 FDEGs (ie *SERPINA3*, *FCN3*, *NPPA*, *CCL2* and *PLA2G2A*) were all involved in the immune system. All of these 5 genes except *NPPA* were down‐regulated in the majority of conditions (Table 2). Briefly, these results indicated that immune systems were suppressed in the progression of HF.

### 3.4 Gene set enrichment analysis reveals dysregulated biological pathways in HF

**Figure 4:**
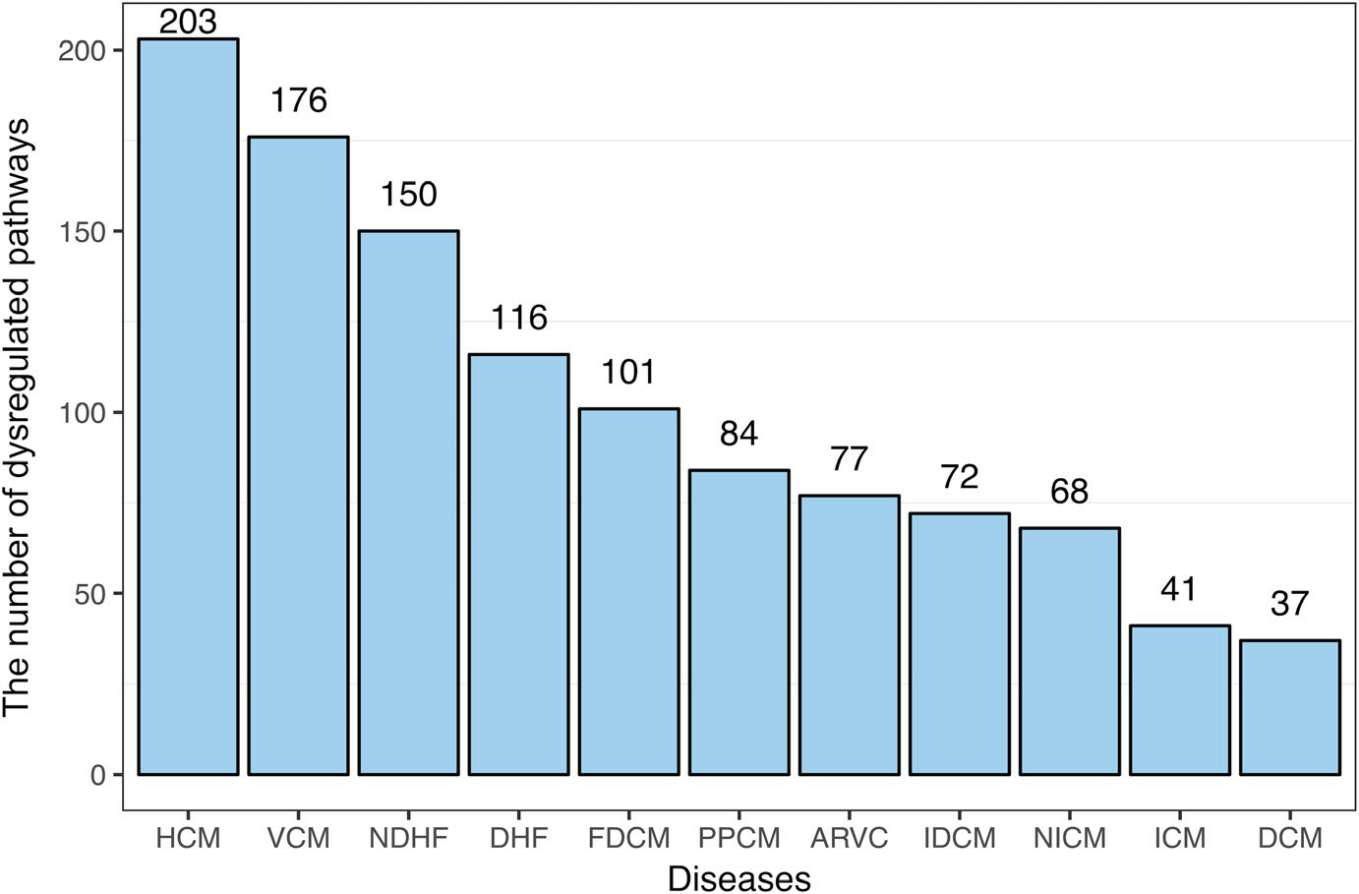
The distribution of dysregulated pathways across HF from 11 different aetiologies. The number above each histogram refers to the number of dysregulated pathways in HF from the corresponding aetiology

To identify the biological pathways that were frequently influenced by HF from different aetiologies, we obtained 1329 curated pathways from the MSigDB database.^14^ After excluding pathways that were too large or too small, overlapping pathways and disease‐related pathways,^26^ 610 pathways were kept for further analysis (see Materials and Methods section). Pathway enrichment analysis of these curated pathways performed with GSEA helped us to obtain a *P*‐value per pathway per condition. GSEA is a computational method that identifies gene sets (eg biological pathways) that show a statistically significant, concordant difference between two biological states.^17^ Based on GSEA, we found that 610 pathways were all dysregulated in at least one of the 414 disease samples (Table S4). A pathway was defined as a dysregulated pathway in HF from a given aetiology if it was identified as significant by GSEA in more than half of the disease samples from the given aetiology. Figure 4 shows the distribution of the dysregulated pathways in HF from 11 different aetiologies. On average, approximately 102 dysregulated pathways were identified in HF from each aetiology. HF from HCM resulted in the maximum number (203) of dysregulated pathways, whereas HF from DCM resulted in the minimum number (37) of dysregulated pathways. This showed that the number of dysregulated pathways changed among the different aetiologies of HF.

**Figure 5:**
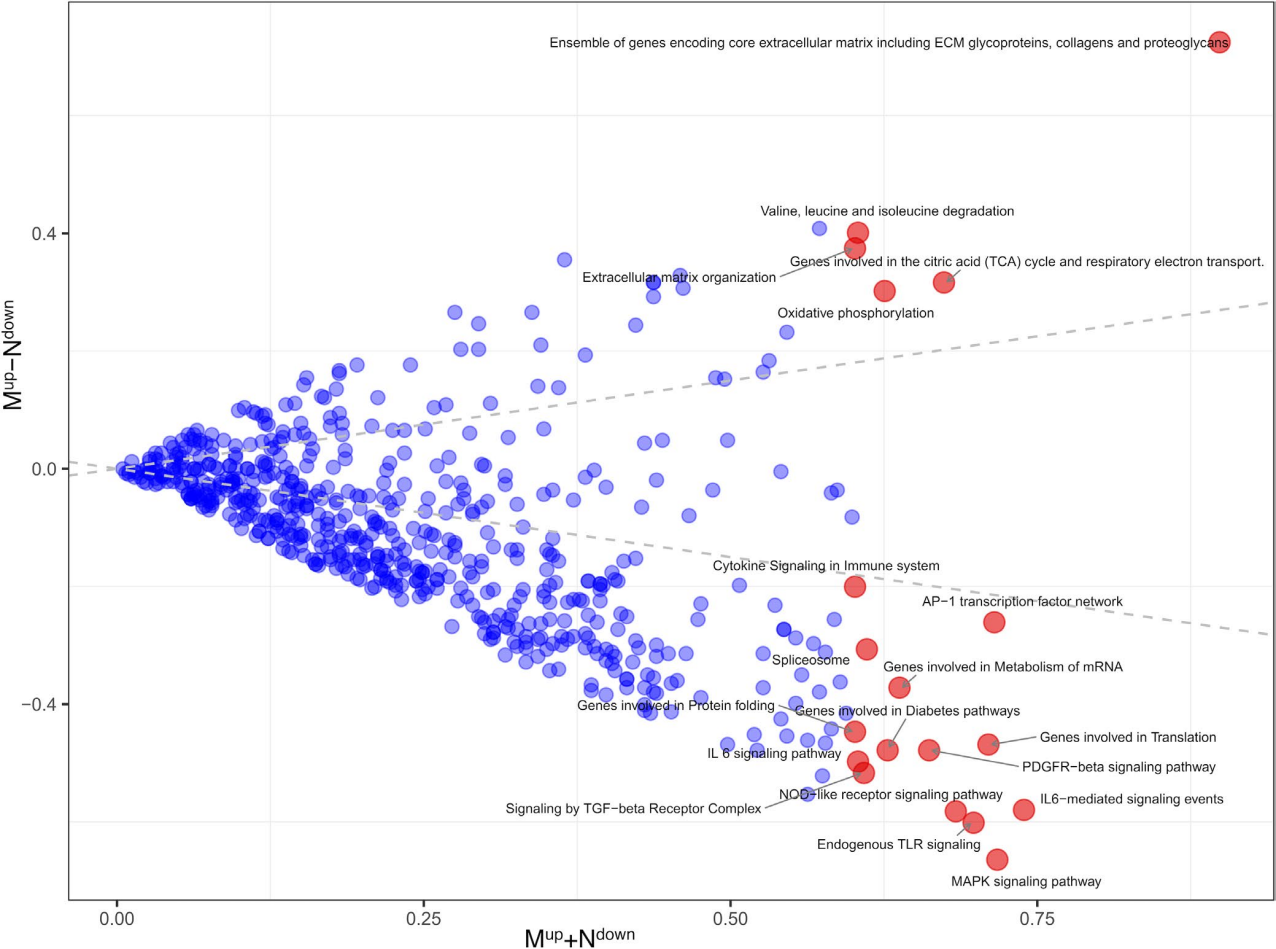
The expression pattern of 610 curated pathways in HF from different aetiologies. Each node represents a pathway, and 19 frequently dysregulated pathways are coloured in red. The *x*‐axis and *y*‐axis are M^up^ + N^down^ and M^up^‐N^down^, respectively, where M^up^ and N^down^ represent the proportion of disease samples in which a given pathway is significantly up‐regulated and down‐regulated, respectively. The dashed lines demarcate the region where the absolute value of N^up^ − N^down^ is < 50% of N^up^ + N^down^ and are generated for visualization purposes only

To identify the pathways that were frequently dysregulated in HF from different aetiologies, we selected pathways that were dysregulated in more than 60% of the HF samples^27^ and get 19 frequently dysregulated pathways in Table 3. As shown in Figure 5, HF from different aetiologies resulted in the consistent up‐regulation or down‐regulation of the majority of frequently dysregulated pathways. These results demonstrated that HF from different aetiologies was associated with some common pathways. The top three significant pathways were the ‘ensemble of genes encoding core extracellular matrix including ECM glycoproteins, collagens and proteoglycans’, ‘IL6‐mediated signalling events’ and the MAPK signalling pathway’ (Table 3). The pathway of the ‘ensemble of genes encoding core extracellular matrix including ECM glycoproteins, collagens and proteoglycans’ was up‐regulated in more than 80% (336/414) of the disease samples. The pathways ‘MAPK signalling pathway’ and ‘IL6‐mediated signalling events’ were down‐regulated in 69% (286/414) and 66% (273/414) of disease samples, respectively. All three pathways played significant roles in cardiac remodelling during various cardiac diseases, such as HF.^28^, ^29^, ^30^, ^31^ When we look deeply into the 19 frequently dysregulated pathways, we found eight pathways involved in the immune system, namely ‘IL6‐mediated signalling events’, ‘MAPK signalling pathway’, ‘AP‐1 transcription factor network’, ‘endogenous TLR signalling’, ‘NOD‐like receptor signalling pathway’, ‘PDGFR‐beta signalling pathway’, ‘cytokine signalling in immune system’ and ‘IL 6 signalling pathway’. Meanwhile, two of the 19 frequently dysregulated pathways were related to the extracellular matrix, namely the ‘ensemble of genes encoding core extracellular matrix including ECM glycoproteins, collagens and proteoglycans’ and ‘extracellular matrix organization’. Moreover, five pathways, that is ‘genes involved in translation’, ‘genes involved in the citric acid (TCA) cycle and respiratory electron transport’, ‘oxidative phosphorylation’, ‘valine, leucine and isoleucine degradation’ and ‘genes involved in metabolism of mRNA’, were involved in metabolism. These results suggested that pathways related to immune system signalling, the extracellular matrix and metabolism might be critical in the development of HF.

**Table 3:** The 19 frequently dysregulated pathways in HF from 11 different aetiologies

Pathway ID	Pathway name	#Significant	#Up	#Down
NABA_CORE_MATRISOME	Ensemble of genes encoding core extracellular matrix including ECM glycoproteins, collagens and proteoglycans	372	336	36
PID_IL6_7_PATHWAY	IL6‐mediated signalling events	306	33	273
KEGG_MAPK_SIGNALING_PATHWAY	MAPK signalling pathway	297	11	286
PID_AP1_PATHWAY	AP‐1 transcription factor network	296	94	202
REACTOME_TRANSLATION	Genes involved in Translation	294	50	244
PID_TOLL_ENDOGENOUS_PATHWAY	Endogenous TLR signalling	289	20	269
KEGG_NOD_LIKE_RECEPTOR_SIGNALING_PATHWAY	NOD‐like receptor signalling pathway	283	21	262
REACTOME_TCA_CYCLE_AND_RESPIRATORY_ELECTRON_TRANSPORT	Genes involved in the citric acid (TCA) cycle and respiratory electron transport.	279	205	74
PID_PDGFRB_PATHWAY	PDGFR‐beta signalling pathway	274	38	236
REACTOME_METABOLISM_OF_MRNA	Genes involved in metabolism of mRNA	264	55	209
REACTOME_DIABETES_PATHWAYS	Genes involved in diabetes pathways	260	31	229
KEGG_OXIDATIVE_PHOSPHORYLATION	Oxidative phosphorylation	259	192	67
KEGG_SPLICEOSOME	Spliceosome	253	63	190
REACTOME_SIGNALING_BY_TGF_BETA_RECEPTOR_COMPLEX	Signalling by TGF‐beta receptor complex	252	19	233
KEGG_VALINE_LEUCINE_AND_ISOLEUCINE_DEGRADATION	Valine, leucine and isoleucine degradation	250	208	42
BIOCARTA_IL6_PATHWAY	IL 6 signalling pathway	250	22	228
REACTOME_CYTOKINE_SIGNALING_IN_IMMUNE_SYSTEM	Cytokine signalling in immune system	249	83	166
REACTOME_PROTEIN_FOLDING	Genes involved in protein folding	249	32	217
REACTOME_EXTRACELLULAR_MATRIX_ORGANIZATION	Extracellular matrix organization	249	202	47

Note

‘#Significant’, ‘#Up’ and ‘#Down’ represent the number of disease samples in which the corresponding pathway was differentially expressed, up‐regulated and down‐regulated, respectively.

### 3.5 Potential TFs regulating frequently dysregulated pathways

**Figure 6:**
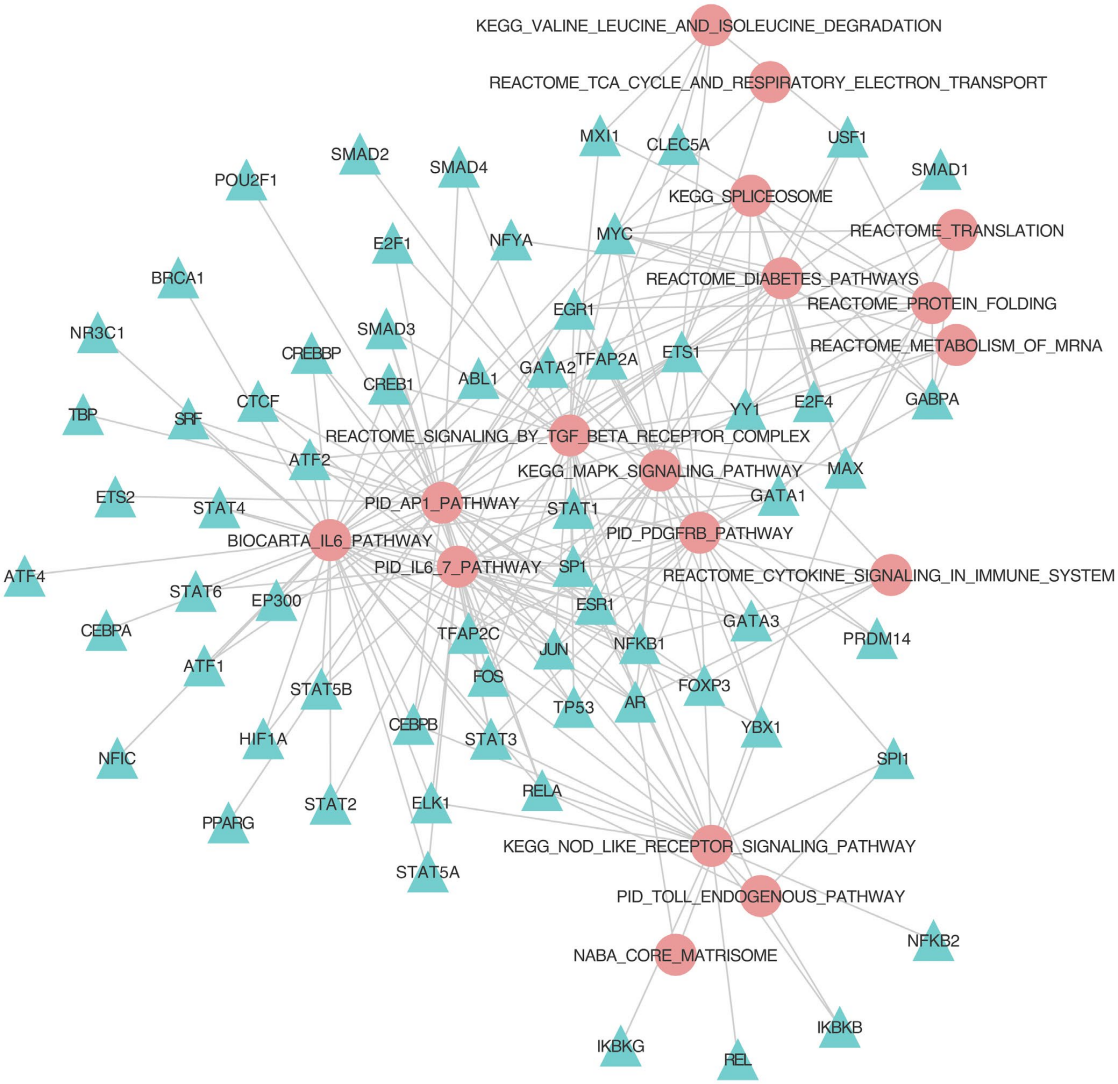
The 241 regulatory relationships between 64 TFs and 17 frequently dysregulated pathways. Circle and triangle nodes represent frequently dysregulated pathways and TFs, respectively. TF‐pathway regulatory relationships were predicted using Fisher's exact test

To gain an in‐depth understanding of how TFs regulated the expression of the 19 frequently dysregulated pathways, we collected 179 785 regulatory interactions from RegNetwork and HTRIdb, which comprised 1438 TFs and 18 396 target genes. Then, the regulatory relationships between the TFs and pathways were tested using Fisher's exact test based on 179 785 regulatory interactions (see Materials and Methods section for details). Given a BH‐corrected *P*‐value threshold of 0.05 and a ratio cut‐off of 0.2, we obtained 241 regulatory relationships between 64 TFs and 17 frequently dysregulated pathways (Table S5, Figure 6). On average, each pathway was predicted to be regulated by approximately 14 TFs, and each TF regulated four pathways. The top 10 TFs with the largest number of degree were *ETS Proto‐Oncogene 1* (*ETS1*), *MYC*, *Specific Protein 1* (*SP1*), *Early Growth Response 1* (*EGR1*), *Nuclear Factor Kappa B Subunit 1* (*NFKB1*), *Yin And Yang 1* (*YY1*), (*Androgen Receptor*) *AR*, *Tumor Protein p53* (*TP53*), (*Transcription Factor AP‐2 Alpha*) *TFAP2A* and *Jun Proto‐Oncogene* (*JUN*), and their detail information is listed in Table S6. These genes may serve as a vital role in regulating these dysregulated pathways and potentially affect the initiation and progression of HF.

## 4 DISCUSSION

With the development of high‐throughput ‘omics’ technologies, large‐scale transcriptional data of HF are available in public databases, providing a good opportunity to analyse HF by integrating these data. Although a few studies were carried out to decipher the molecular mechanisms of HF from different aetiologies,^6^, ^7^ these mechanisms have not previously been analysed by integrating large‐scale transcriptional profiles and pathway information. More importantly, the molecular commonality of HF from different aetiologies was unclear. In this study, we integrated transcriptional profiles and pathway information to investigate the molecular commonalities of HF from 11 different aetiologies.

Some previous analyses explored HF by using transcriptional data and interaction networks.^6^, ^7^, ^32^, ^33^, ^34^, ^35^, ^36^ However, using small‐scale transcriptional data and interaction networks with high positive rates may lead to constrained results. In this work, we performed a three‐tiered transcriptional data analysis by integrating large‐scale transcriptional data and curated pathways, which can produce more solid results. The advantages of integrating curated pathways include reducing the complexity by grouping thousands of DEGs into just several hundred pathways and increasing the explanatory power by identifying impacted curated pathways with specific functions.

Our approach not only successfully uncovered several key genes (such as *NPPA*, *NPPB* and *FRZB*) already involved in HF but also provided new candidate genes involved in HF for further experimental verification. The signal transducer and activator of transcription (STAT) family contains seven members (*STAT1*, *STAT2*, *STAT3*, *STAT4*, *STAT5*, *STAT5B and STAT6*), and all genes have been reported to be expressed in the heart.^37^ Five of the seven *STAT* genes (*STAT1*, *STAT2*, *STAT3*, *STAT5* and *STAT6*) were reported to play roles in regulating the progression of HF.^38^, ^39^ The role of *STAT4* in HF has not been reported, but it was up‐regulated in 20 HF conditions in this analysis. Moreover, *STAT4* was predicted to regulate two dysregulated pathways (Figure 6). Considering the importance of *STAT* genes in HF and the consistent up‐regulation of *STAT4* in HF from different aetiologies, it was reasonable to suggest that *STAT4* may play an important role in HF. *SERPINA3*, a protease inhibitor, was found to be down‐regulated in the failing myocardium from patients with DCM,^40^ and the up‐regulation of *SERPINA3* is associated with poor survival in patients with HF.^41^ In this work, a meta‐analysis found that *SERPINA3* was down‐regulated in all HF conditions, which further confirmed the importance of *SERPINA3* in HF. It is possible that *SERPINA3* might become a novel diagnostic and therapeutic target for HF. Lucas et al found that gene *osteoglycin (OGN)* is overexpressed in patients with HF and proposed that *OGN* can act as a potential biomarker for ischaemic HF.^42^ In this work, *OGN* is not only overexpressed in ischaemic HF but also overexpressed in non‐ischaemic HF. Thus, the role of *OGN* in non‐ischaemic HF needs further investigation.

Several frequently dysregulated pathways identified from GSEA, for example the ‘MAPK signalling pathway’, ‘valine, leucine and isoleucine degradation’ and ‘ensemble of genes encoding core extracellular matrix including ECM glycoproteins, collagens and proteoglycans’, have already been implicated in HF. The MAPK signalling pathway consists of a well‐studied family of serine/threonine proteins that include the extracellular signal‐regulated protein kinases (ERKs), the c‐Jun N‐terminal kinases (JNKs) and the p38 family of kinases. ERKs,^43^ JNKs^44^ and p38 MAP kinase^45^ are all involved in HF. Leucine, isoleucine and valine belonging to the branched‐chain amino acids (BCAA) represent the most abundant group of essential amino acids that cannot be synthesized de novo.^46^ BCAA catabolic deficiency was proposed as a novel metabolic feature in HF with a broad impact on the progression of pathological remodelling and dysfunction.^47^ Multiple studies have already shown HF‐related changes in cardiac ECM, including the accumulation in glycoproteins, collagens and proteoglycans.^48^ We noticed that a disease pathway ‘genes involved in diabetes pathways’ was also identified as a frequently down‐regulated pathway (Table 3). HF is closely related to diabetes: patients with HF are at higher risk of developing diabetes.^49^ The enrichment of canonical pathway ‘genes involved in diabetes pathways’ in HF further confirmed the close connection between HF and diabetes. In addition, our analysis also discovered several biological pathways with potential roles in HF from different aetiologies, for example the ‘PDGFR‐beta signalling pathway’. Chintalgattu et al found that PDGFR‐beta knock‐out mice exposed to load‐induced stress resulted in HF and showed that cardiomyocyte PDGFR‐β signalling plays a vital role in stress‐induced cardiac angiogenesis.^50^ It is reasonable to speculate that the PDGFR‐beta signalling pathway may regulate angiogenesis in the heart, which substantially contributes to HF through several different mechanisms.

It has been long recognized that immune system activation or dysregulation plays a significant role in the development and progression of HF.^51^ In this work, both gene‐centric differential expression analysis and pathway‐centric enrichment analysis revealed that immune system‐related genes and pathways were significantly changed in HF from 11 different aetiologies. Annotation analysis showed that down‐regulated FDEGs were significantly enriched in the GO terms ‘inflammatory response’ and ‘defence response’. Moreover, the top 5 FDEGs, namely *SERPINA3*, *FCN3*, *NPPA*, *CCL2* and *PLA2G2A*, were all involved in the immune system (Table 2). GSEA identified several pathways frequently dysregulated in HF from 11 aetiologies, and approximately 42% (8/19) of the frequently dysregulated pathways were involved in the immune system (Table 3). These results further confirmed the importance of the immune system in HFs and that their role in HF was independent of the aetiologies of HF.

Generally, TFs play key roles in regulating the expression of encoding genes and tend to regulate genes within the same pathways. In this study, we also predicted 64 potential TFs regulating 17 dysregulating pathways. Some predicted TFs, such as *TP53* and *NFKB1*, have already been reported involving in the progression of HF *TP53* was proven to be a master regulator of the cardiac transcriptome and a key molecule, which triggered the development of HF.^52^, ^53^ *NFKB* is a pleiotropic TF involved in different signalling pathways and strongly implicated in the development of cardiac remodelling, hypertrophy and HF.^54^, ^55^, ^56^ *NFKB1* belongs to the NFKB TF family, and it has been reported that *NFKB1* polymorphism is associated with the heart function in patients with HF from different aetiologies.^57^ The other predicted TFs with unknown roles in HF are good candidates for further experimental verification, such as *ETS1* and *EGR1*. It is known that *ETS1* is important in heart development. Moreover, a previous study found that patients with congenital heart disease had a de novo frameshift mutation in *ETS1*.^58^ In the present study, we found that *ETS1* is predicted to regulate the expression of fourteen dysregulating pathways. We speculated that *ETS1* participated in the progression of HF by regulating the expression of these dysregulated pathways. *EGR1* is an early‐response TF that can be rapidly induced by various environmental stimuli. It was predicted to regulate 8 dysregulated pathways and identified as a FDEG (Table S2). In the previous studies, *EGR1* was found to involve in multiple cardiovascular pathobiology including cardiac hypertrophy, atherosclerosis, ischaemic pathology and angiogenesis.^59^ Furthermore, the expression level of *EGR1* can discriminate between chronic HF patients and control patients.^59^ Therefore, we speculated that *ETS1* might be a potential biomarker of HF.

Finally, we recognized some limitations in this work. Our results are based on currently available data and should be interpreted with caution. First, our analyses are limited by the availability of pathway and gene expression information. Therefore, some genes with potential roles in HF are ignored in this work, as these genes are not detected on the microarray or included in the curated pathways. Second, the analysis of transcriptomes is often not enough to reflect the level of pathway activity, this weaken the conclusions that can be drawn from our results. Third, further molecular biological experiments are needed to confirm the function of these key genes and TFs, and how they involve in the progression of HF.

In summary, we performed a three‐tiered transcriptional data analysis to explore the molecular commonalities of HF from different aetiologies. Our analyses indicate that HF from different aetiologies is associated with 111 FDEGs and 19 frequently dysregulated pathways. It is hoped that our current analyses can provide new insight to understand the molecular mechanisms of HF from different aetiologies.

## CONFLICTS OF INTEREST

The authors declare that they have no conflicts of interest.

## AUTHOR CONTRIBUTION

**Zhenhong Jiang:** Data curation (lead); Formal analysis (lead); Methodology (lead); Visualization (lead); Writing‐original draft (lead); Writing‐review & editing (lead). **Ninghong Guo:** Data curation (supporting); Formal analysis (supporting); Methodology (supporting); Writing‐original draft (supporting); Writing‐review & editing (supporting). **Kui Hong:** Conceptualization (lead); Funding acquisition (lead); Project administration (lead); Supervision (lead); Writing‐original draft (supporting); Writing‐review & editing (supporting).

## Supporting information

Fig S1

Table S1

Table S2

Table S3

Table S4

Table S5

Table S6
