# Supplementary figures and images for: A three‐tiered integrative analysis of transcriptional data reveals the shared pathways related to heart failure from different aetiologies
Source: J Cell Mol Med. 2020 Jul 8;24(16):9085–96. doi: 10.1111/jcmm.15544 (PMC7417717; doi:10.1111/jcmm.15544)

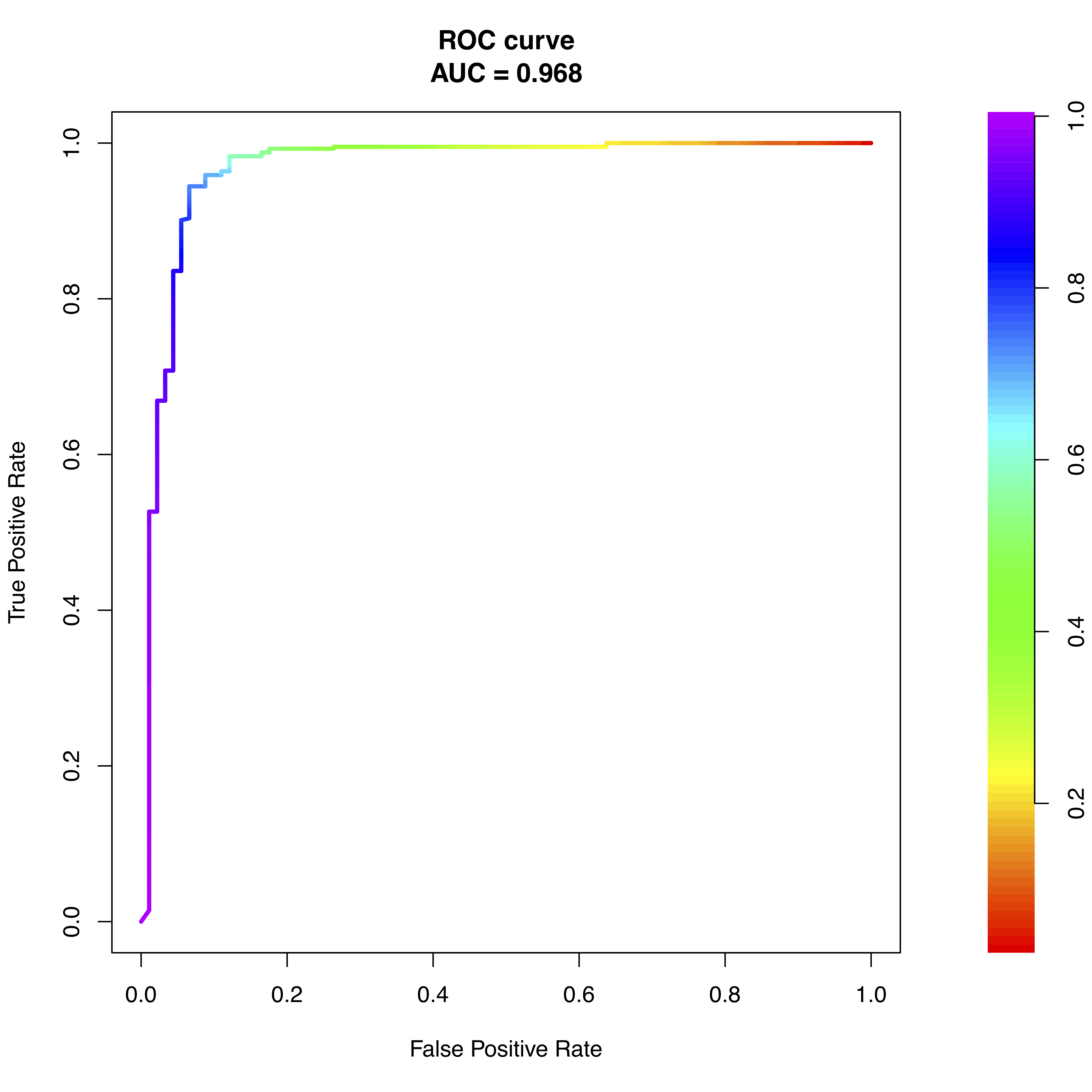

Supplement: Supplementary file 1 — Fig S1 [file JCMM-24-9085-s001.tiff]
